# A French national observatory of people with HIV initiating lenacapavir-based treatment after regulatory approval

**DOI:** 10.1128/aac.00228-26

**Published:** 2026-06-10

**Authors:** Charlotte Charpentier, Thomas Montrouge, Marc Wirden, Véronique Avettand-Fenoel, Camille Tumiotto, Pantxika Bellecave, Audrey Rodallec, Elisabeth Garnier, Djeneba Fofana, Sidonie Lambert-Niclot, Brigitte Montès, Marie-Laure Chaix, Caroline Charre, Pierre Gantner, Magali Bouvier-Alias, Enagnon Kazali Alidjinou, Pauline Coulon, Gilbert Mchantaf, Agnès Beby-Defaux, Elodie Alessandri-Gradt, Alice Moisan, Quentin Le Hingrat, Minh Lê, Constance Delaugerre, Anne-Geneviève Marcelin, Gilles Peytavin, Vincent Calvez, Diane Descamps, the Lenacapavir Observatory Study Group, Kevin Alexandre

**Affiliations:** 1 Service de Virologie, AP-HP, Hôpital Bichat-Claude Bernard, Université Paris Cité, INSERM, IAME 555089, Paris, France; 2 Service de Virologie, Sorbonne Université, INSERM, UMR-S 1136, Institut Pierre Louis d'Epidémiologie et de Santé Publique, AP-HP, Hôpitaux Universitaires Pitié Salpêtrière-Charles Foix 55577 , Paris, France; 3 LI2RSO, CHU d’Orléans, Service de Virologie, Université d’Orléans, Orléans, France; 4 Service de Virologie, CHU de Bordeaux, CNRS-UMR 5234 Microbiologie Fondamentale et Pathogénicité, Université de Bordeaux, Bordeaux, France; 5 CHU Nantes, Service de Virologie, Nantes, France; 6 Département de Virologie, Santé Sorbonne Université, INSERM, Institut Pierre Louis d'Epidémiologie et de Santé Publique APHP, Hôpital Saint-Antoine 243485, Paris, France; 7 Service de Virologie, CHU de Montpellier 26905 , Montpellier, France; 8 Service de Virologie, INSERM UMR 1342, AP-HP, Hôpital Saint-Louis, Université Paris Cité 555089, Paris, France; 9 Service de Virologie, CHU de Strasbourg 36604 , Strasbourg, France; 10 Service de Virologie, AP-HP, Hôpital Henri Mondor 378967 , Créteil, France; 11 Laboratoire de Virologie ULR3610, Univ Lille, CHU de Lille 26902, Lille, France; 12 Service de Virologie, CHU de Poitiers 36655 , Poitiers, France; 13 Department of Virology, National Reference Center of HIV, Univ Rouen Normandie, Université de Caen Normandie, INSERM, Normandie Univ, DYNAMICURE UMR 1311, CHU Rouen, Rouen, France; 14 Service de Pharmacologie, AP-HP, Hôpital Bichat-Claude Bernard, INSERM, IAME 55076, Paris, France; Chinese Academy of Medical Sciences & Peking Union Medical College, Beijing, China

**Keywords:** cabotegravir, drug resistance, lenacapavir, HIV

## Abstract

There is limited data on lenacapavir (LEN) use, the newest capsid inhibitor, in observational settings. We describe population characteristics and pharmaco-virological outcomes of people with HIV-1 (PWH) who initiated LEN-based treatment. We conducted a national retrospective observational study of PWH initiating LEN-based treatment in France after its approval (December 2022). Virological failure (VF) was defined as two consecutive viral loads (VLs) ≥50 c/mL, and a non-virological response as a VL decrease of <1 log_10_ c/mL or still >50 c/mL at W24. Ninety-six PWH were included; 49 were virologically suppressed at initiation. Median follow-up on LEN was 12 months (IQR = 8–17). Genotypic susceptibility score was <1 in 59 cases (61%). Twelve participants (12.5%) discontinued LEN-based treatment. Among the virologically suppressed and viremic PWH at initiation, 94% and 64% had VL <50 c/mL at the last follow-up visit, respectively. VF occurred in 8 PWH (3 in virological success and 5 viremic at baseline), and a non-virological response was observed in 12 PWH. Capsid sequence at VF was available for eight subjects, showing the emergence of N74D mutation in one. LEN plasma concentrations were available for 13 of the 20 PWH presenting with VF or non-response with adequate concentrations in 90% of cases. Twenty-four participants received cabotegravir + LEN, 18 having VL <50 c/mL at the last follow-up visit. Our observational findings confirm that LEN-based regimens are effective among heavily treatment-experienced individuals with advanced resistance. In this population, LEN-based treatments were associated with high rates of sustained virological suppression and a low incidence of capsid emergent resistance.

## INTRODUCTION

Lenacapavir (LEN) is the first-in-class ARV capsid inhibitor, targeting multiple steps of HIV replication, including capsid assembly, nuclear import, and genome packaging (1). LEN has a prolonged half-life (8–12 weeks) after oral loading dose and subcutaneous (SC) administration, with limited potential for drug interactions due to low hepatic clearance (2, 3). Pivotal phase 2/3 CAPELLA trial demonstrated the efficacy and durability of LEN added to an optimized background therapy (OBT) in people with HIV-1 (PWH) heavily treatment-experienced (HTE) with multidrug-resistant (MDR) viruses (4–7). The trial achieved a virological suppression rate of 61.4% at Week 156 (W156) (7). Of the 28 participants meeting criteria for genotypic resistance test (GRT), emergence of LEN resistance mutations was reported in 14 participants (4–7). It is worth noting that LEN was used as functional monotherapy in all 14 cases, due to the absence of active ARV associated with LEN, or to non-adherence to active oral drugs associated with LEN (4–7).

Real-world evidence on the use of LEN remains scarce. Preliminary data from the French Compassionate Use Program, which included 33 HTE individuals with MDR HIV-1 viruses, showed a virological suppression rate of 67% at W26, supporting its effectiveness in routine care despite complex resistance profiles (8).

Given the limited availability of observational data, additional studies are warranted to better characterize participants’ profiles, treatment combinations, virological response, and pharmacological data under real-life conditions. The aim of this study was to describe the population characteristics and pharmaco-virological outcome of PWH initiating a LEN-based regimen in a real-world setting.

## RESULTS

### Participants’ characteristics

Ninety-six PWH from 14 French clinical centers were included. Overall, the median age was 57 years (IQR = 43–62), and 64% (*n* = 61) were men. Median CD4 count nadir was 66 cells/mm^3^ (IQR = 14–142) and median viral load (VL) zenith was 5.6 log_10_ c/mL (IQR = 5.2–5.9). HIV-1 subtype was B in 57 subjects (59%), CRF02_AG in 15 (16%), and other subtypes/CRFs in 24 (25%). An historical HIV-1 tropism was available for 61 participants, being R5 in 31 cases (51%) and X4 in 30 cases (49%).

**Fig 1:**
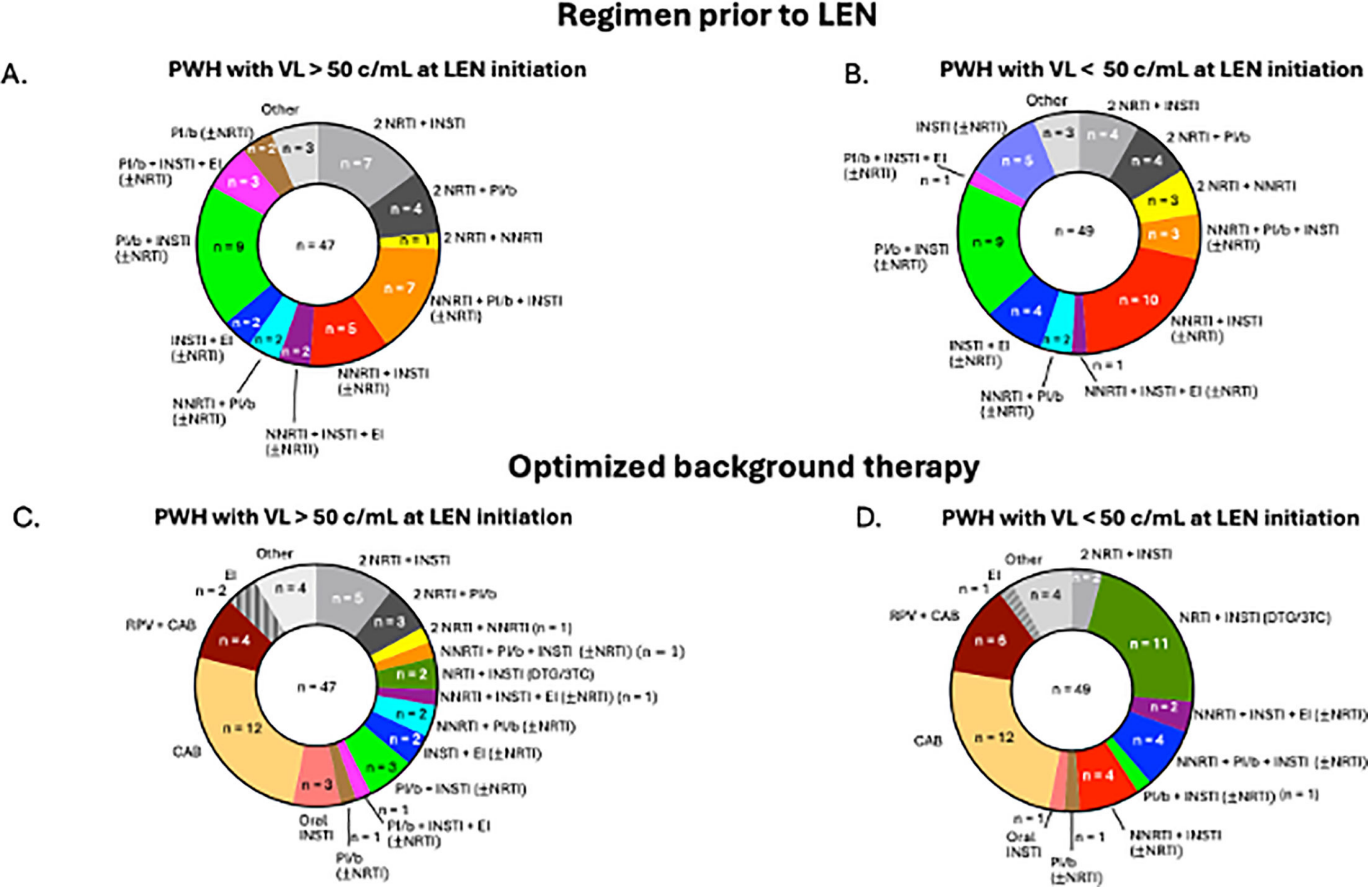
Description of the major antiretroviral drug classes included in the regimen prior to the lenacapavir-based regimen (**A** and **B**) and of the optimized background therapy (**C** and **D**) depending on viral load at LEN initiation. 3TC, lamivudine; CAB, cabotegravir; EI, entry inhibitors; INSTI, integrase strand-transfer inhibitor; LEN, lenacapavir; NNRTI, non-nucleoside RT inhibitor; NRTI, nucleoside RT inhibitor; PI/b, boosted protease inhibitor; PWH, people with HIV; RPV, rilpivirine.

At LEN + OBT initiation, median CD4 cell count was 395**/**mm^3^ (IQR = 162–621), and 49 (51%) participants were in virological suppression (i.e., VL < 50 c/mL). Among participants in treatment failure at initiation (*n* = 47, 49%), median VL was 2.4 log_10_ c/mL (IQR = 1.9–3.3). Overall, ARV regimens prior to LEN + OBT were very heterogeneous; the median number of ARV included in the regimen was 3 (IQR = 3–4), with the most common combinations being two nucleoside reverse transcriptase (RT) inhibitors (NRTIs) + integrase strand-transfer inhibitor (INSTI) (*n* = 11, 11%), and two NRTI + INSTI + boosted/PI (*n* = 12, 13%) (Fig. 1A and B).

### Cumulative resistance at LEN + OBT initiation

**Fig 2:**
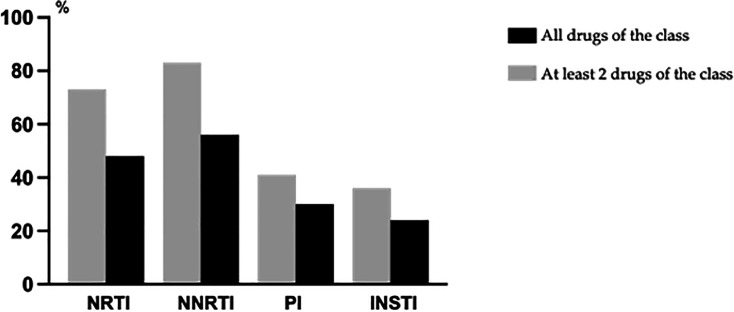
Percentage of participants with viruses resistant to at least two drugs of an antiretroviral drug class (gray light bars) or resistant to all drugs of the antiretroviral drug class (black bars).

The participants had a median of five historical GRTs (IQR = 3–9), median duration since the first genotype being 14 years (IQR = 8–20). Overall, the mean number of drug resistance mutations was 4 (range = 0–9), 3 (range = 0–6), 2 (range = 0–6), and 1 (range = 0–5) for NRTI, NNRTI, PI, and INSTI, respectively. The percentage of participants having a virus being resistant to all drugs in each class was 48%, 56%, 30%, and 24% for NRTI, NNRTI, PI, and INSTI, respectively (Fig. 2). The percentage of participants having a virus resistant to at least two drugs in each class was 73%, 83%, 41%, and 36% for NRTI, NNRTI, PI, and INSTI, respectively (Fig. 2). Overall, 52% of participants have two or more fully exhausted ARV drug classes. The most frequent NRTI, NNRTI, major PI, and INSTI resistance mutations were M184V (*n* = 66, 69%), Y181C/V (*n* = 38, 40%), M46I/L (*n* = 33, 34%), and N155H (*n* = 15, 16%), respectively (Fig. S1). Historical gp41 sequences were available for 23 participants showing resistance to the fusion inhibitor enfuvirtide in five cases. Historical gp120 sequences were available for 16 participants showing polymorphisms associated with resistance to the attachment inhibitor fostemsavir in two cases.

### Optimized background therapy and pharmaco-virological outcome

The median number of ARV in the OBT was 2 (IQR = 1–3), with INSTI being the most frequently used (81% of cases) (Fig. 1C and D). Since participants were HTE with MDR viruses, 13 (14%) were also receiving fostemsavir. The dual long-acting strategy including the INSTI cabotegravir (CAB) and LEN was received by 24 participants (25%). Overall, the GSS, based on cumulative historical genotypes, was ≤1 in 58 participants (60%).

The median follow-up on LEN-based treatment was 12 months (IQR = 8–16). Twelve PWH discontinued LEN-based treatment (12.5%): three lost to follow-up, four for virological failure (VF), one for cutaneous side effects, one participant’s decision, and three deaths unrelated to LEN.

Among the 49 virologically suppressed PWH at LEN + OBT initiation, 46 (94%) had VL <50 c/mL at the last follow-up visit and 3 (6%) had a VF. Among the 47 viremic PWH at LEN + OBT initiation, 30 (64%) reached VL <50 c/mL at the last follow-up visit, 5 (11%) had a VF, and 12 (26%) a non-virological response (Table 1). The characteristics of the 20 participants in VF or in non-response are described in Table 2. The median GSS of participants in virological success was 1 (IQR = 1–2) and was 1.75 (IQR = 1.0–2.5) for those in VF or in non-response. For the eight patients with VF, the first time point of VF occurred in median at 6 months (range = 1.2–10.7) after LEN initiation with a median VL of 156 c/mL (IQR = 101–258). The median VL at the last time point visit among the 12 non-responders participants was 219 c/mL (IQR = 115–395).

**TABLE 1:** Virological outcome at last follow-up visit, depending on HIV viral load at LEN + OBT initiation, at the virological suppression thresholds of 50 and 200 c/mL^*a*^

	Virological outcome at last follow-up visit	Baseline VL < 50 /mL *n* = 49	Baseline VL > 50 cmL *n* = 47	Overall *n* = 96
Threshold 50 c/mL	Virological success	46 (94 %)	30 (64 %)	76 (79 %)
	Virological failure	3 (6 %)	5 (11 %)	8 (8 %)
	Non-response	–^*b*^	12 (26 %)	12 (13 %)
Threshold 200 c/mL	Virological success	48 (98 %)	37 (79 %)	85 (89 %)
	Virological failure	1 (2 %)	2 (4 %)	3 (3 %)
	Non-response	–	8 (18 %)	8 (8 %)

^*a*^ LEN, lenacapavir; OBT, optimized background therapy; VL, viral load.

^*b*^ –, not applicable.

**TABLE 2:** Description of the 20 participants in virological failure or presenting a non-virological response during the follow-up^*a*^

Participant ID	Age (years)	Gender	HIV-1 subtype	Regimen prior to LEN	VL at LEN initiation (c/mL)	Optimized background therapy	GSS	Virological outcome	Time point at failure (months)	VL at failure (c/mL)	Capsid sequence at failure	Protease, RT, integrase sequences at failure
1	55	M	URF	2 NRTI + PI/b + INSTI	246	TAF/FTC/BIC	1.5	Non-response	6	206	WT	No additional mutation
2	74	F	CRF02_AG	2 NRTI + PI/b	296	TDF/FTC + DRV/b	2	Non-response	6	52	Not performed	Not performed
3	65	F	B	2 NRTI + INSTI	87	CAB	1	Non-response	6	72	Not amplified	Not amplified
5	77	F	B	NRTI + INSTI	142	DTG/3TC	1	Non-response	6	94	Not amplified	Not amplified
8	34	M	F1	2 NRTI + PI/b	1770	TDF/FTC + DRV/b	2	Non-response	6	22,100	Not amplified	No additional mutation
9	39	F	CRF13_cpx	2 NRTI + PI/b + INSTI	209,000	TAF/FTC/EVG /c + DRV	2.5	Non-response	8	1420	WT	No additional mutation
10	32	F	URF	2 NRTI + PI/b	49,600	TDF/FTC + DRV/b	3	Non-response	1	316	N74D	No additional mutation
11	59	M	B	NRTI + NNRTI + PI/b	164	DRV/b	1	Non-response	6	122	Not performed	Not performed
12	72	M	B	PI/b + INSTI + MVC + FTR	120	DRV/b + DTG + FTR	2.5	Non-response	8	286	Not amplified	Not performed
14	33	F	B	2 NRTI + PI/b + INSTI	382,345	DTG/3TC + FTR	2	Non-response	7	232	WT	No additional mutation
17	32	F	B	NRTI + NNRTI + INSTI	251	DOR + DTG + FTR	2.5	Non-response	18	631	Not amplified	No additional mutation
19	58	M	B	NRTI + PI/b	98	CAB	1	Non-response	8	204	WT	No additional mutation
4	46	M	CRF02_AG	NRTI + NNRTI + PI/b	139	CAB	1	VF	8	90	Not amplified	Not amplified
7	71	F	CRF02_AG	NRTI + PI/b + ENF	798	TDF/FTC + DRV/r + ENF	4	VF	6	265	Not amplified	Not amplified
15	43	F	CRF02_AG	2 NRTI + PI/b	80	TDF/FTC	1	VF	6	80	WT	No additional mutation
16	58	F	CRF02_AG	2 NRTI + NNRTI	119,000	CAB	1	VF	6	255	Not amplified	No additional mutation
20	54	F	CRF02_AG	2 NRTI + INSTI + MVC	86	CAB	1	VF	4	121	Not amplified	Not amplified
6	56	M	CRF02_AG	2 NRTI + PI/b + INSTI	<50	RPV + CAB + MVC	3	VF	1	105	Not amplified	No additional mutation
13	69	M	B	NRTI + NNrTI + PI/b + INSTI	<50	DTG/3TC + MVC	2	VF	11	191	WT	R5 to X4 switch
18	66	M	B	PI/b + INSTI + MVC	<50	CAB	1	VF	7	604	WT	No additional mutation

^*a*^ 3TC, lamivudine; BIC, bictegravir; CAB, cabotegravir; DOR, doravirine; DRV/b, boosted darunavir; DTG, dolutegravir; ENF, enfuvirtide; EVG/c, elvitegravir boosted with cobicistat; F, female; FTC, emtricitabine; FTR, fostemsavir; GSS, genotypic susceptibility score; INSTI, integrase strand-transfer inhibitor; M, male; MVC, maraviroc; NNRTI, non-nucleoside RT inhibitor; NRTI, nucleoside RT inhibitor; PI/b, boosted protease inhibitor; RPV, rilpivirine; TAF, tenofovir alafenamide fumarate; TDF, tenofovir disoproxil fumarate; VF, virological failure; VL, viral load; WT, wild-type.

Overall, at the threshold of VL <200 c/mL, 85 participants (89%) achieved or maintained virological suppression. Among the 11 individuals with VL above 200 c/mL, all but one were viremic at LEN + OBT initiation, k8 had a non-virological response profile, and 3 experienced a VF (Table 1).

Regarding the 24 participants receiving dual long-acting therapy CAB + LEN, half (*n* = 12) were viremic at initiation and half were virologically suppressed. At the last time of follow-up, 18 (75%) had VL <50 c/mL (4 experienced a VF and 2 a non-virological response), increasing to 21 (88%) at the VL <200 c/mL threshold. Of the six participants with VL >50 c/mL at the last follow-up visit, five were viremic at CAB + LEN initiation.

### Genotypic resistance and pharmacological analysis

A capsid sequence was obtained for 8 of the 20 PWH with VL >50 c/mL at the last follow-up visit (5 non-virological response and 3 VF). Emergence of LEN resistance mutation was observed in one case (5.3%). Among the 12 participants with available protease, RT, or integrase sequences at failure, no emergence of new resistance mutations to the OBT drugs was observed. An R5-to-X4 tropism switch was observed in one participant receiving maraviroc.

**Fig 3:**
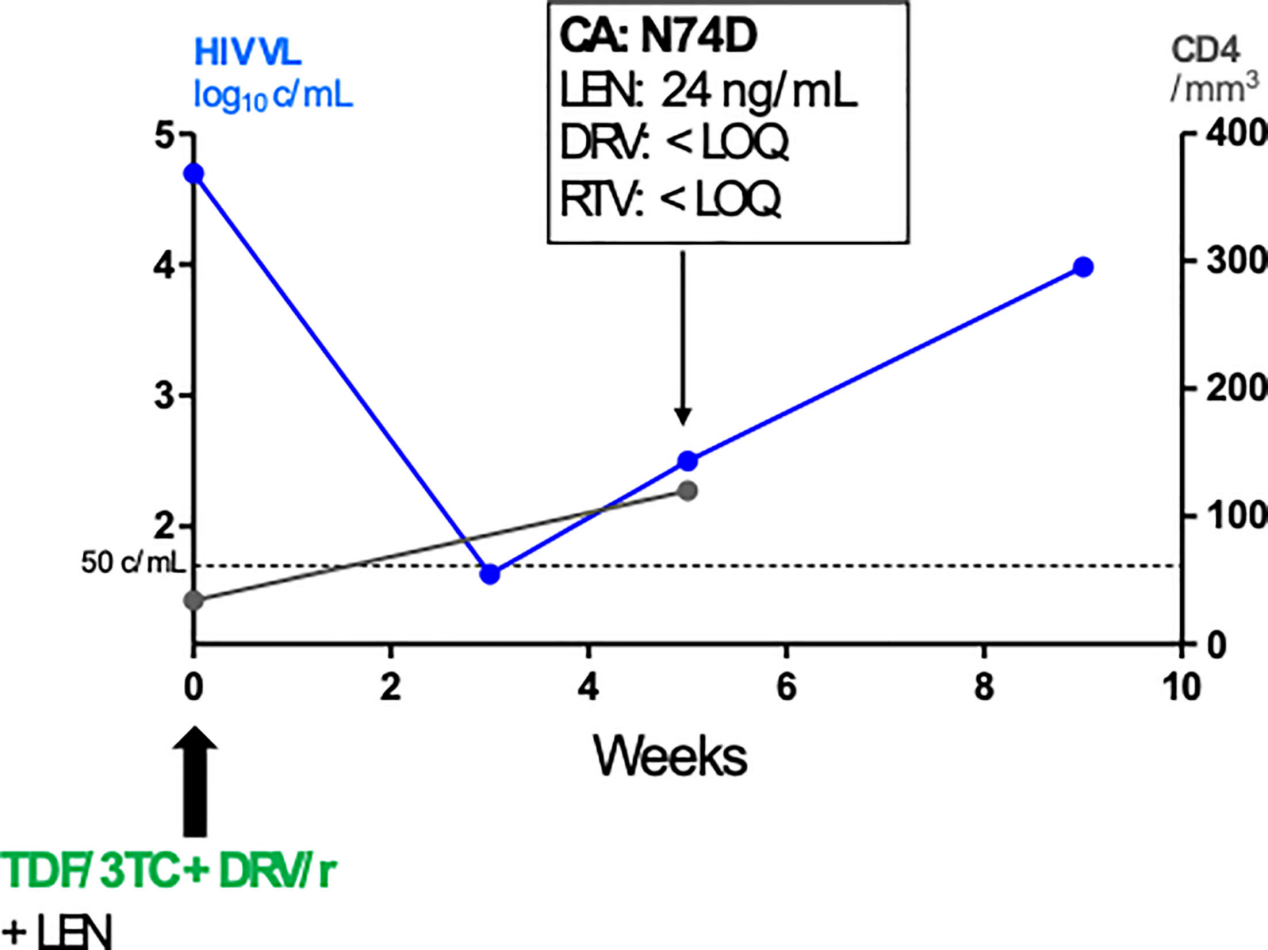
Description of the follow-up of the participant with emergence of resistance to lenacapavir at time of virological failure.

The only participant with a LEN resistance mutation emerging at VF (N74D mutation) had an initial virological response, with VF occurring early at W5 of LEN + OBT (Fig. 3), with a VL of 316 c/mL. He was subsequently lost to follow-up after W9. This participant had a GSS of 3, since the three drugs of the OBT were active (i.e., tenofovir disoproxil fumarate, lamivudine, and ritonavir [RTV]-boosted darunavir [DRV]). However, DRV and RTV trough plasma concentrations were below the LOQ evidencing suboptimal adherence to oral OBT leading to LEN functional monotherapy (Fig. 3).

**Fig 4:**
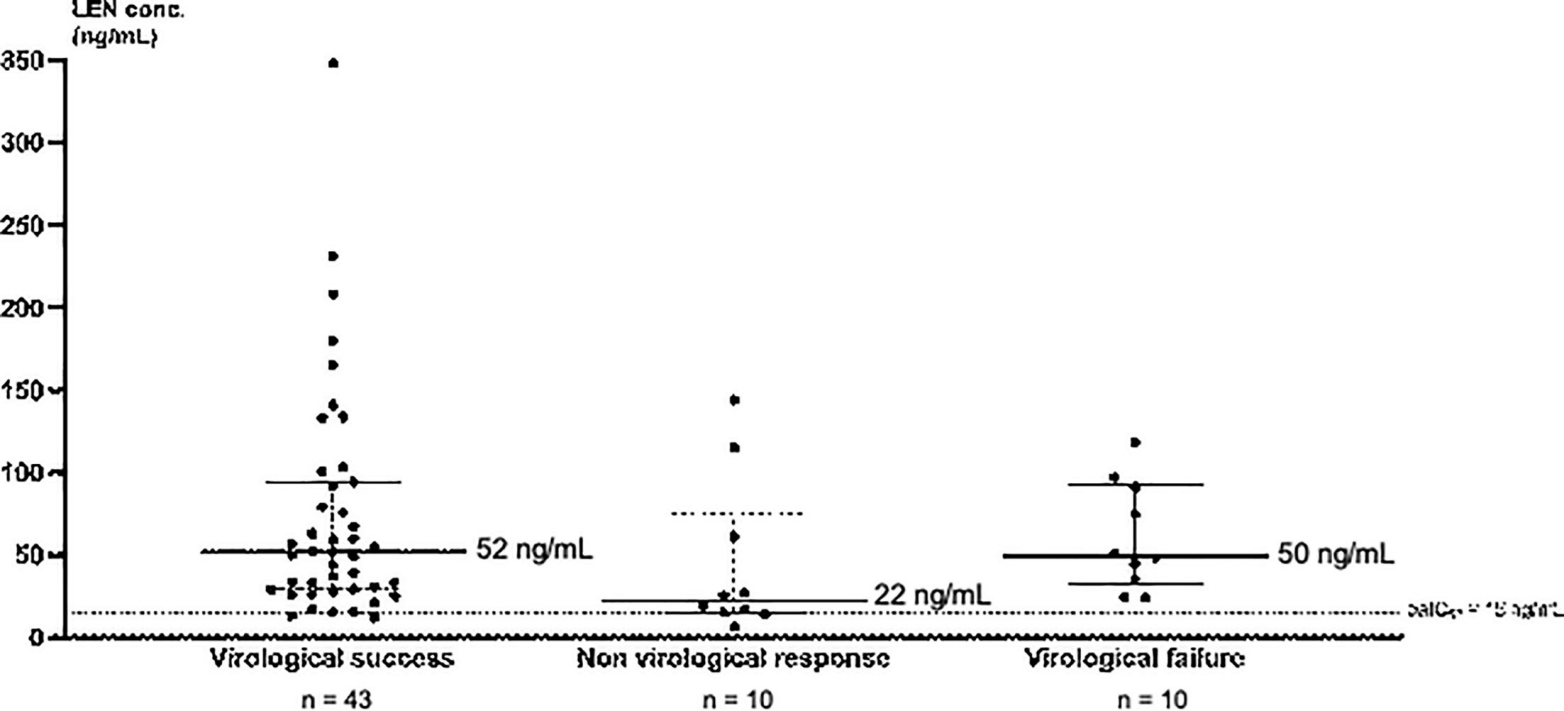
Distribution of the lenacapavir plasma concentrations obtained 180 days (±30) post-injection depending on the virological outcome. Median and interquartile ranges are indicated.

Regarding pharmacological analysis, at least one LEN plasma level measure was available at Day 180 (±30 days) post-injection for six individuals with a non-response profile and seven with a VF, showing adequate LEN plasma concentrations in 18 out of 20 measurements (90%). Adequate OBT plasma concentrations were shown in three of the four participants with a non-virological response or a VF. Among participants in virological success, at least one LEN plasma level measure was available for 29, showing adequate LEN concentrations in 41 of the 43 analyzed samples (95%) (Fig. 4).

**Fig 5:**
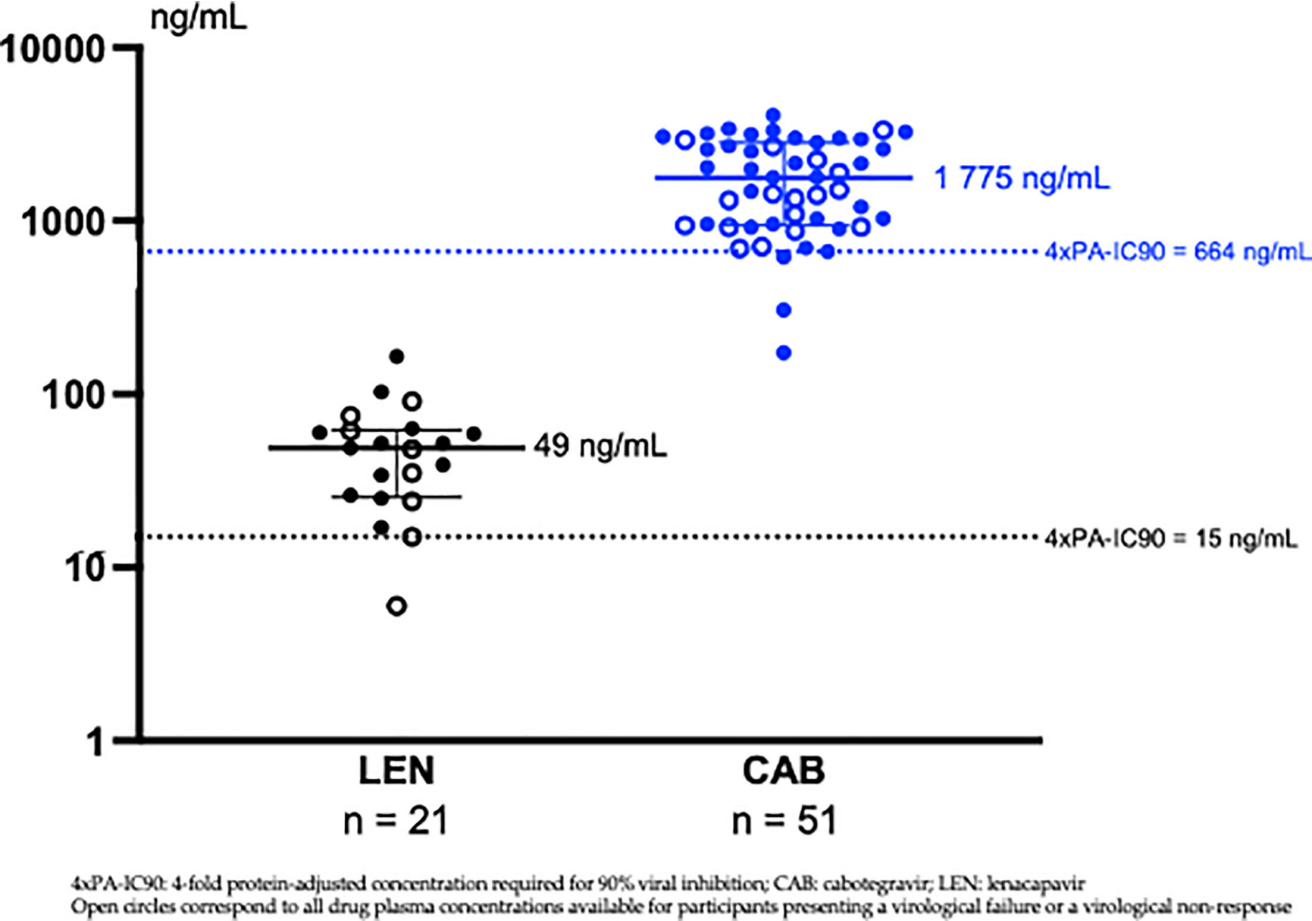
Distribution of the lenacapavir (LEN) and cabotegravir (CAB) plasma concentrations obtained 180 days (±30) or 30 days (±10) post-injection, respectively, among participants receiving CAB + LEN, for whom concentrations of both drugs were available. Median and interquartile ranges are indicated. 4xPA-IC90, 4-fold protein-adjusted concentration required for 90% viral inhibition; CAB, cabotegravir; LEN, lenacapavir. Open circles correspond to all drug plasma concentrations available for participants presenting a virological failure or a virological non-response.

At least one plasma drug concentration was available for 15 individuals receiving CAB + LEN (9 with virological success, 3 with non-virological response, and 3 with VF) representing 51 CAB concentrations at Day 60 (±10) post-injection and 21 LEN concentrations at Day 180 (±30) post-injection. All LEN plasma concentrations, but one, were adequate (≥15 ng/mL) and 48 out of 51 CAB concentrations were adequate (≥664 ng/mL) (Fig. 5).

## DISCUSSION

This study is the first to provide a description of LEN use outside of a clinical trial following regulatory approval in France. Among the 96 PWH with MDR viruses and long, complex treatment histories, a high level of virological suppression was achieved during the follow-up.

In the present study, approximately half of the participants were virologically suppressed at LEN-based regimen initiation, and LEN was introduced in most cases to simplify their ART regimen. Virological suppression was maintained in 94% of them at last follow-up, highlighting the efficacy of LEN-containing regimens in this population. Among viremic participants at LEN + OBT initiation, virological suppression (<50 c/mL) was achieved in 64% of cases at 12 months in median, a rate comparable to the 61% observed in the CAPELLA trial at the later time point of W156 (7). These results align with those of the French Compassionate Use Program, in which 53% and 74% of viremic participants achieved VL < 50 c/mL by W26 and W52, respectively (8). Taken together, these data suggest that LEN-based regimen remains effective in real-life conditions, despite a high level of resistance and OBT heterogeneity. Furthermore, we observed a high virological suppression rate of 79% among viremic participants at LEN + OBT initiation at the 200 c/mL threshold, confirming the efficacy of LEN, even in individuals who have exhausted multiple ARV classes.

Almost two-thirds (61%) of participants in the present cohort had an overall GSS ≤ 1, reflecting severely limited treatment options. Nevertheless, virological success was achieved in 64% of viremic participants at initiation, confirming that LEN + OBT can achieve virological suppression even in the presence of partial resistance to OBT. Use of fostemsavir in 14% of participants and maraviroc in a few cases illustrates the importance of designing the OBT individually based on the interpretation of cumulative resistance.

In our study, among the 20 participants experiencing VF or virological non-response, LEN resistance was observed in only one case out of eight with successful sequencing (12.5%). This favorably contrasts with the results of the CAPELLA trial in which LEN resistance mutations were observed in 50% of participants experiencing failure (4–7). One possible explanation for this difference could be the level of VL at VF, as the median VL level at VF in our cohort is only 156 c/mL. The sole case of LEN resistance in our cohort, among the available genotypes at failure, occurred in the context of poor adherence to active oral ARV therapy, resulting in functional LEN monotherapy. This finding is consistent with previous reports that have linked LEN resistance emergence to inadequate adherence to OBT (4–7, 9). Indeed, in all participants in the CAPELLA trial with emergence of LEN resistance, LEN was in functional monotherapy due to non-adherence to oral drugs or no active ARV associated (4–7). These findings emphasize that, as previously observed with other ARV agents, non-adherence and inactive OBT remain the major drivers of LEN resistance. In the present study, only one case of resistance to OBT drugs was observed, in a participant who experienced a tropism switch from R5 to X4 while receiving maraviroc.

Importantly, a quarter of our cohort received a dual long-acting regimen combining LEN and CAB, representing one of the very few observations of this therapeutic strategy. Recently, it has been described that, among 10 viremic PWH receiving CAB + LEN ± rilpivirine, there was a mean decrease of 3.88 log_10_ c/mL within a median of 2 months (10). In the present study, we observed virological suppression rates of 75% and 88% at thresholds of 50 and 200 c/mL, respectively. These results support the feasibility of this combination in selected individuals.

Among all participants of this study with available LEN plasma levels, 94% of samples showed adequate concentrations, confirming the expected pharmacokinetic profile under real-life conditions. As expected, a high inter-patient variability was observed. Our data also showed that most participants presenting with VF or a non-virological response had adequate LEN plasma concentrations. Among participants receiving CAB + LEN with available pharmacokinetics data, we observed a very high percentage with adequate plasma concentrations of both drugs, confirming the feasibility of this dual long-acting combination.

This study presents the first multicenter, national data set on the use of LEN after approval in France, supported by detailed virological and pharmacological data. However, several limitations should be acknowledged, notably its retrospective design, the heterogeneity of follow-up intervals, and the limited availability of genotypic resistance testing and pharmacological data of the OBT at failure.

In conclusion, our observational study confirms the effectiveness of LEN-based regimens among individuals with advanced resistance who have undergone multiple treatments. LEN + OBT was shown to achieve high rates of sustained virological suppression, low incidence of emergent resistance, provided treatment adherence, and maintained OBT activity. The data also highlight the growing interest in long-acting regimens combining LEN with other agents such as CAB, which may broaden the therapeutic landscape for PWH with complex resistance or adherence barriers. Future efforts should focus on the prospective monitoring of resistance emergence as well as integrating pharmacological data and long-term outcome analysis, with the aim of optimizing LEN-based strategies in routine care.

## MATERIALS AND METHODS

### Study participants and virological outcome definition

This is a French national retrospective observational study including PWH initiating a LEN-based treatment in centers from the French National Agency of Research on AIDS (ANRS|MIE) virology and pharmacology network after LEN approval in France (22 December 2022). All participants were HTE with at least one GRT showing drug resistance mutations to at least one ARV of two major drug classes. A VF was defined as two consecutive viral loads (VLs) of ≥50 c/mL, regardless of the baseline VL. Non-virological response was defined as a decrease in VL of <1 log₁₀ c/mL or failure to achieve virological suppression (VL < 50 c/mL) at W24 for those who were viremic at baseline.

Participants initiated usual or simplified regimen combining oral loading tablets (1,200 mg) and SC dose (927 mg) then SC-maintenance dose (927 mg) every 6 months. For those receiving CAB + LEN containing regimen, CAB 600 mg intramuscular injection (IM) was administered at Day 0 and W4, then Q8W to synchronize the injections with those of LEN. The length of the needle used for CAB IM was adjusted according to body mass index (>30 kg/m^2^).

### Data collection and virological outcome definition

Immuno-virological and therapeutic status at baseline were collected. Historical RNA GRT were collected and cumulative GRT were interpreted using the ANRS|MIE algorithm (https://hivfrenchresistance.org/). Major protease inhibitors (PIs) resistance mutations were defined according to the IAS-USA resistance mutations list (11). Genotypic susceptibility scores (GSS), with scores of 1 for susceptibility, 0.5 for possible resistance, and 0 for full resistance, were determined using the ANRS|MIE. The overall susceptibility score of the OBT was the sum of individual scores. RT, protease, integrase, and capsid regions were sequenced in each laboratory of the ANRS|MIE Virology & PK network. Three different methods were used: ANRS|MIE protocol (www.hivfrenchresistance.org), Abbott ViroSeq kit, or an in-house method.

Plasma concentrations of LEN and other ARV were determined by UPLC-MS/MS (Waters Acquity, Milford, MA, USA) with LOQ < 5 ng/mL. Plasma concentrations of CAB and LEN were determined at W8 and W26 and were interpreted using a threshold of 664 and 15 ng/mL, respectively, corresponding to fourfold human serum protein-adjusted 95% effective concentration on wild-type HIV-1 (,^12^, ^13^).

## Data Availability

The data that support the findings of this study are available from the corresponding author upon reasonable request.

## SUPPLEMENTAL MATERIAL

Fig. S1 aac.00228-26-s0001.docx Description of the drug resistance mutations detected in the cumulative resistance genotypes derived from plasma viruses of the 96 participants.

ASM does not own the copyrights to Supplemental Material that may be linked to, or accessed through, an article. The authors have granted ASM a non-exclusive, world-wide license to publish the Supplemental Material files. Please contact the corresponding author directly for reuse.
