# Supplementary material for: A French national observatory of people with HIV initiating lenacapavir-based treatment after regulatory approval
Source: Antimicrob Agents Chemother. 2026 Jun 10;70(7):e00228-26. doi: 10.1128/aac.00228-26 (PMC13321793; doi:10.1128/aac.00228-26)
Supplement: Fig. S1 — Description of the drug resistance mutations detected in the cumulative resistance genotypes derived from plasma viruses of the 96 participants. [file aac.00228-26-s0001.docx]

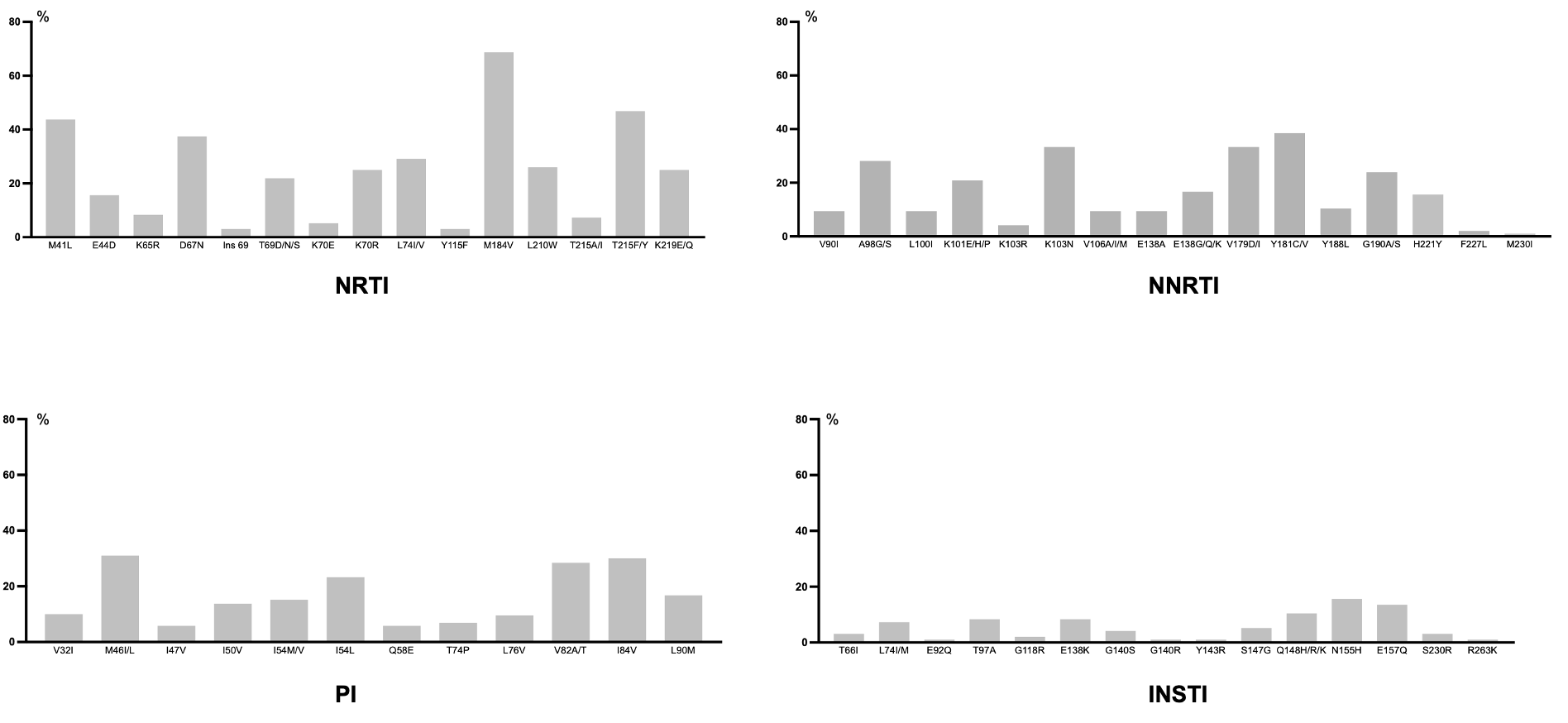
**Supplementary figure 1.** Description of the drug resistance mutations detected in the cumulative resistance genotypes derived from plasma viruses of the 96 participants.

INSTI: integrase strand-transfer inhibitor; NNRTI: non-nucleoside RT inhibitor; NRTI: nucleoside RT inhibitor; PI: protease inhibitor.
